# (In)COGNITO: Unmasking Factors Driving Divergence From Guideline-Directed Management of Cardiac Implantable Electronic Device Infections∗

**DOI:** 10.1016/j.jacadv.2023.100770

**Published:** 2023-12-20

**Authors:** Andrew H. Voigt, Saketram Komanduri, Krishna Kancharla

**Affiliations:** University of Pittsburgh Medical Center, Pittsburgh, Pennsylvania, USA

**Keywords:** CIED infection, lead extraction, survey

**Figure:**
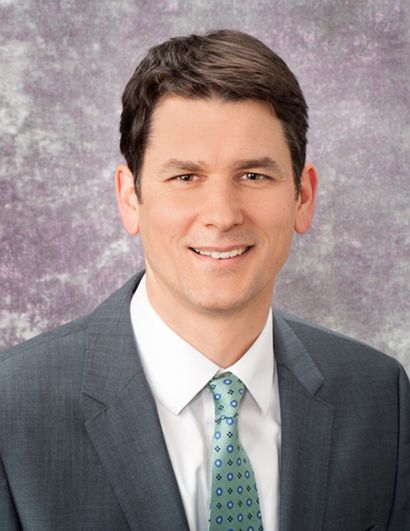

Utilization of cardiac implantable electronic devices (CIEDs) continues to grow in the context of an aging population with complex medical comorbidities. There has been an increase in both implantation and the rate of infections in the last few decades.^1^ Several expert consensus documents provide CIED infection management best practices based on the available evidence.^2^^,^^3^ Current guidelines provide a Class I recommendation for complete CIED extraction, in addition to antibiotic therapy, to improve morbidity and mortality. How well have we been doing?

Recent data have been sobering. An analysis of the Nationwide Readmissions Database by Sciria et al^4^ suggested that only 11.5% of patients identified as having a CIED and endocarditis received transvenous lead extraction from 2016 through 2019. Advanced age, renal disease, admission to smaller hospitals, female sex, and dementia were all associated with lower utilization of CIED extraction in this study. Lead extraction was independently associated with improved mortality. An analysis by Lee et al suggested an increased in-hospital mortality associated with delayed (>7 days) lead extraction for patients with CIED infections.^5^ What factors influence appropriate management decisions by physicians caring for patients with CIED infections?

In this issue of *JACC: Advances*, Birgersdotter-Green et al^6^ describe the results of COGNITO (Contemporary Management of Cardiac Implantable Electronic Device Infection: A Survey of American College of Cardiology Members and Primary Care Physicians), a survey administered to U.S. physicians in early 2022 to gain insight into the real-world management of CIED infection. We congratulate the authors and the American College of Cardiology for this effort to uncover factors that may be contributing to the low utilization of lead extraction in CIED infection management despite a Class I recommendation in the practice guidelines.

The survey is structured with a questionnaire and case-based examples to understand the core knowledge of guidelines and physician practice patterns. The response rate was low (20%), perhaps typical of a voluntary survey. The survey included responses from 387 physicians from a broad geographical area across the United States and multiple practice settings and with varying clinical experience. The study participants included 35% electrophysiologists, 46% non-electrophysiology cardiologists, and only 19% primary care physicians. Notably, this is the largest societal-based survey to understand practice patterns in CIED infection management across a spectrum of physician backgrounds.

The survey finds a strikingly low rate of familiarity with the current practice guidelines for CIED infection management among non-electrophysiology cardiologists (29%) and primary care physicians (23%) compared to electrophysiologists (91%). Only 30% of respondents reported that a guideline-based CIED infection protocol/pathway existed at their hospital. When presented with clinical vignettes in which the current guidelines would recommend CIED system removal, electrophysiologists tended to follow these guidelines more frequently (89%) than cardiologists (50%) or primary care physicians (30%). Perhaps it is not surprising that electrophysiologists reported more familiarity with the latest practice guidelines in this area, as compared to general cardiologists and primary care physicians. In a similar way, a survey evaluating the management of dyslipidemia or coronary artery disease would surely favor the knowledge of general and interventional cardiologists over electrophysiologists. The survey results do, however, urge us to utilize the expertise of specialized resources with electrophysiology and infectious diseases consultation to manage CIED infections and, thus, improve guidelines adherence and patient outcomes.

COGNITO demonstrates not just differential knowledge and expertise but a gulf between perception and reality. Twenty percent of physician respondents perceive either high or very high risk (>6%) of major complications related to lead extraction, which is higher than the 2% risk that published data suggest.^7^ The study reports that the perceived risk of the lead extraction procedure was one of the 2 major decision factors to consider for referral for lead extraction. Approximately 40% of electrophysiologists who do not perform lead extraction reported managing device infection without a referral for extraction. The survey findings suggest an underutilization of referrals for lead extraction, perhaps in part due to the perceived high risk of significant complications from the procedure.

While lead extraction is associated with a risk of life-threatening complications such as venous tear and pericardial effusion, such risk is moderate based on the literature from a national database.^7^ The risk of sepsis, endocarditis, and related mortality in patients with incompletely treated CIED infection may be much higher.^8^ However, it is essential to acknowledge that lead extraction and reimplantation are associated with procedural risk, significant hospital time, and patient morbidity and inconvenience. We should remain open to utilization of approaches to minimize hospital time, such as using temporary devices in the interim prior to reimplantation. For example, for patients with a primary prevention implantable cardioverter-defibrillator, guidelines support the usage of a wearable defibrillator prior to reimplantation.^9^ Leadless pacemaker systems and subcutaneous implantable cardioverter-defibrillators decrease the risk of infection and should be considered when appropriate.^10^^,^^11^ With advanced age, complex comorbidities, and frailty, some patients may be at prohibitive risk for lead extraction procedures and may benefit from multidisciplinary review. Alternative approaches such as long-term suppressive antibiotic therapy should be considered based on factors such as bacterial cultures, antibiotic sensitivity, and patient tolerability. High-volume centers like ours utilize a multidisciplinary team involving endovascular service, infectious disease, electrophysiology, cardiothoracic surgery, addiction medicine, cardiology, and a primary patient care team for risk stratification and comprehensive management of CIED infections. Availability of such resources at an institutional level is paramount for adequate management.

Several significant study limitations bear emphasis. With such a low survey completion rate and a low percentage of primary care physicians participating, it is unclear to what extent a true cross section of physicians participated. There is potential selection bias as survey respondents may have self-selected as physicians interested in CIED management. Finally, the clinical scenarios presented are oversimplified versions of real-world practice. The clinical vignettes do not adequately account for factors such as patient age, frailty, age of leads, pacemaker dependence, and other clinical factors that influence medical decision-making in patients with CIED infection.

## How do we respond to the study results? a call to action

*“Failure is not fatal, but failure to change might be”*

—John Wooden

While there are limitations to the current study, it suggests that low awareness of guideline-directed recommendations for CIED infection and overestimation of risks associated with lead extraction (with attendant limitations on appropriate referral) probably both contribute to suboptimal management of patients with CIED infection. Underutilization of lead extraction has been associated with higher morbidity and mortality. Now it is time for action. The response should be considered at multiple levels. Often, the guidelines and best practices from subspecialty areas do not adequately reach the practitioners in other disciplines. Creating awareness locally through institutional pathways is essential to improve early referral patterns and utilize a multidisciplinary approach to specialized care. Electronic medical record-based alert notifications for referral and incentives for appropriate referral at the institutional level can help educate and motivate physicians for guideline-directed practice. Cardiovascular and heart rhythm societies should partner with their medical and surgical counterparts to disseminate the benefits of guideline adherence and best practices to all practitioners. Ultimately, our patients are central to all management decisions. It remains crucial to educate patients regarding signs of infection, monitor device sites for erosions, and seek immediate assistance from the team caring for the patient, including the electrophysiologist. Appropriate care for patients with CIED infection mandates multidisciplinary care and shared decision-making.

Birgersdotter and coauthors’ findings should encourage us to reflect on the state of affairs in CIED infection management. Lead extraction may indeed have an image problem in the wider medical and cardiology communities. These perceptions may be limiting appropriate referrals and care. More institutions need a clear pathway for infection management. The study calls for education to address the gaps in knowledge and further efforts to streamline care and referral pathways for appropriate management of patients with CIED infection.

## Funding support and author disclosures

Dr Kancharla has industry relationships with Boston Scientific-Education and Varian Medical Systems-DSMB. All other authors have reported that they have no relationships relevant to the contents of this paper to disclose.
